# Sex-related change in BMI of 15- to 16-year-old Norwegian girls in cross-sectional studies in 2002 and 2017

**DOI:** 10.1186/s12887-019-1790-2

**Published:** 2019-11-12

**Authors:** Asborg A. Bjertnaes, Jacob H. Grundt, Petur B. Juliusson, Trond J. Markestad, Tor A. Strand, Mads N. Holten-Andersen

**Affiliations:** 1 Department of Paediatric and Adolescent Medicine, Innlandet Hospital Trust, Anders Sandvigs gate 17, 2609 Lillehammer, Norway; 2 Department of Clinical Medicine, University of Oslo, Oslo, Norway; 3 Department of Paediatrics, Oslo University Hospital, Oslo, Norway; 4 Department of Health Registries, Norwegian Institute of Public Health, Oslo, Norway; 5 Department of Clinical Science, University of Bergen, Bergen, Norway; 6 Department of Paediatrics, Haukeland University Hospital, Bergen, Norway; 7 Department of Research, Innlandet Hospital Trust, Brumunddal, Norway

**Keywords:** Adolescent, Body mass index, Body mass index distribution, Obesity, Overweight, Sex differences

## Abstract

**Background:**

The prevalence of overweight and obesity (OWOB) has stabilized in some countries, but a portion of children with high body mass index (BMI) may have become heavier. This study aimed to describe the distributions of BMI and the point prevalence of OWOB in Norwegian adolescents in 2002 and 2017.

**Methods:**

A cross-sectional study involving 15- to 16-year-old adolescents in Oppland, Norway, was undertaken in 2002 and 2017. We calculated their BMI, BMI z-scores (BMIz), and the prevalence of OWOB.

**Results:**

The mean BMI increased from 20.7 to 21.4 (*p* < 0.001) for girls but remained unchanged at 21.5 vs 21.4 (*p* = 0.80) for boys. The prevalence of OWOB increased from 9 to 14% among girls (difference 5, 95% CI: 2, 8) and from 17 to 20% among boys (difference 3, 95% CI: − 1, 6%). The BMI density plots revealed similar shapes at both time points for both sexes, but the distribution for girls shifted to the right from 2002 to 2017.

**Conclusion:**

Contrary to previous knowledge, we found that the increase in OWOB presented a uniform shift in the entire BMI distribution for 15–16-year-old Norwegian girls and was not due to a larger shift in a specific subpopulation in the upper percentiles.

## Background

The relationship between body mass index (BMI) in adolescence and subsequent health in adulthood is well established [1–3], and both overly low and overly high BMI values are of concern [4]. The prevalence of adolescent overweight and obesity (OWOB) has increased over the last decades [5], and studies have found that this change is primarily due to increasing BMI in subgroups in the upper percentiles of the BMI distribution [6].

Population changes in BMI distributions over time have been studied in many countries [7–9], including the US [10]. However, relatively few European studies have addressed this issue in adolescents, and even fewer are based on data from the last decade when the obesity epidemic is said to have stabilized in some countries [11].

Adolescents with obesity have a high risk of becoming adults with obesity [12]. As both the biology of OWOB [13] and comorbidities due to central fat distribution differ by sex [14], sex-related trends in adolescent OWOB are important to elucidate for public health reasons.

In this study, we compared BMI distributions and the prevalence of OWOB in Norwegian adolescents in 10th grade (15–16 years of age) at 15-year intervals stratified by sex. Our aim was to explore whether an increasing mean BMI and prevalence of OWOB was due to increasing BMI within a subgroup of adolescents.

## Methods

### Subjects

**Fig. 1:**
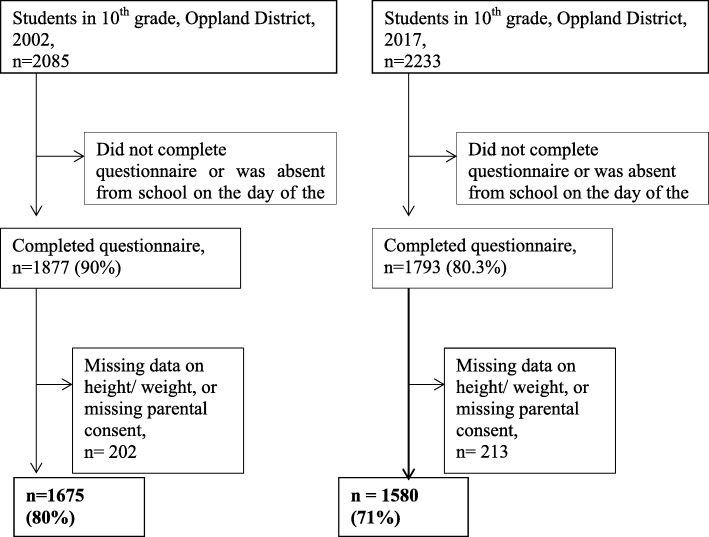
Flowchart

This cross-sectional study was based on questionnaires answered by 10th grade students (15–16 years old) in high schools in the district of Oppland, Norway, in April–June 2002 (*n* = 2085) and in April–May 2017 (*n* = 2233) (Fig. 1). Oppland is one of 18 counties in Norway and had a population of 183,000 in 2002 and 190,000 in 2017. The county is predominantly rural but has two major cities, each with populations of 25,000-30,000.

### Protocol and measures

The Norwegian Institute of Public Health conducted the first survey in 2002 [15]. We conducted the second in 2017 in collaboration with the County Governor of Oppland, the supreme authority of all high schools in the county.

The survey was a paper-questionnaire in 2002 and a web-based questionnaire in 2017. Central questions including health, nutrition, activity, and perceived familial socioeconomic status from 2002, were repeated in 2017. Current weight and height measurements were self-reported in both surveys. The questionnaire used in 2017 was piloted among 842 students in 22 schools in 2015–2016. The 2002 questionnaire lacked the date of height and weight measurements. This date was needed to calculate z-scores; thus, the date of questionnaire completion was used.

### Variables

#### Outcome variables

Anthropometric measurements included self-reported weight (to the nearest kg) and height (to the nearest cm). Based on the self-reported anthropometric data, three outcome variables were calculated: BMI, BMI z-score (BMIz), and OWOB vs. under-weight and normal-weight. For both 2002 and 2017-data, we based BMIz on updated Norwegian growth reference data [16] and defined OWOB according to the International Obesity Task Force (IOTF) [17].

#### Background data (Table 1)

Information on sex and age was available for all participants. The following background data were also collected.

**Table 1:** Descriptive statistics of the background variables

	2002			2017		
	N	%	OWOB %^a^	N	%	OWOB %^a^
Sex	1675	823		1580	814	
Girl	823	49.1	9.0	814	51.5	14.0
Boy	852	50.9	17.0	766	48.5	20.0
Age, years (mean) *SD*	1675	(15.9) *0.3*		1580	(15.8) *0.4*	
Sociodemography of the family						
Not living with siblings	1631	17.0	14.4	1566	16.5	22.1
Poor family economy	1656	3.5	24.1	1572	4.0	23.8
Parents not living together	1664	27.1	13.1	1573	32.1	20.0
Father working full time	1644	84.2	12.2	1560	84.5	16.4
Mother working full time	1654	57.8	11.9	1569	70.2	16.0
Teeth brushing ≤ once daily	1670	25.7	18.4	1573	24.1	24.0
Rural living	1675	74.1	13.0	1580	72.3	18.7
Smoking						
Never smoked	1671	62.4	13.2	1567	87.0	16.3
Parental smoking	1675	35.8	14.0	1580	12.4	23.0
Mental health issues						
Sought help for mental health issues^b^	1573	5.2	14.6	1541	13.0	21.9
Activity						
Spare-time physical activity < 4 times weekly^c^	1570	56.9	14.3	1576	55.9	19.3
Screen time > 2 h/daily	1666	57.6	14.8	1571	68.7	17.6
Participates in organized spare-time sports	1659	43.5	9.3	1563	56.8	12.2
Walking or riding bike to school	1665	35.6	11.6	1577	39.9	14.6
Student education						
Educational plans > 12 years	1659	46.6	11.7	1573	61.0	13.0
Best or second-best grades^d^	1555	55.3	11.2	1506	67.8	14.7
Positive opinion on education^e^	1642	68.9	11.9	1565	78.9	16.2
Nutrition						
Daily breakfast	1673	65.8	13.9	1576	62.9	15.3
Drinking sugar-containing soda ≥ daily	1664	37.5	14.4	1561	13.4	16.3
Eating candy ≥ daily	1660	17.7	9.9	1566	8.2	14.8

^a^% overweight and obesity (OWOB) within the given category

^b^during the last 12 months

^c^activity generating sweating or heavy breathing

^d^in ≥ 1 of 4 subjects: Norwegian writing, mathematics, social science, English

^e^Answered yes to ≥1 of the questions “my education is interesting and I learn a lot”, “good grades are important to me”, and “my parents find education important”

*Socio-demography of the family*: We asked the adolescents if they lived with any siblings, how they classified their family economy compared to other families (poor, average, good, very good), if the parents lived together, and if the parents had full-time employment (full-time/ part-time/ unemployed or receiving social security services/ housewife/ student/ dead). We also included a question on frequency of teeth brushing (< every second day, every second day, once daily, >once daily), as higher family socioeconomic status is associated with greater odds of teeth brushing twice a day or more [18]. As a measure of rural living, place of residence was dichotomized into more or less than 20, 000 inhabitants. *Smoking:* We recorded smoking habits of the adolescents (never, used to but quit, sometimes, daily) and of their parents (yes/no). *Mental health*: We asked the adolescents if they had sought help for mental health problems in the past 12 months (yes/no). *Activity*: We recorded how frequently the adolescents participated in spare-time activities that generated heavy breathing or sweating (never, once per week, 2–3 times per week, 4–6 times per week, daily), how long they watched a screen (phone, computer, TV, tablet) daily during out-of-school hours (< 1 h, 1–2 h, 3–5 h, > 5 h), if they attended organized spare-time sport activities (yes/no) and if they rode a bike or walked to school (yes/no).

We asked the adolescents to describe *student education* by three proxies: educational plans (planning for an education for 9 years, 11 years, 12 years, college or university degree), achievement of good grades (best or second-best grade in ≥1 of the following subjects: Norwegian writing, mathematics, English, social science), and whether they had a positive opinion on education (answered agree/partly agree to ≥one of the three questions “my education is interesting, and I learn a lot”, “good grades are important to me”, and “my parents find education important”). The adolescents also answered questions regarding *nutrition* by reporting how often they ate breakfast (seldom/never, 1–2 times per week, 3–4 times per week, 5–6 times per week, daily), drank sugar-sweetened soda (seldom/never, 1–6 glasses a week, 1 glass daily, 2–3 glasses daily, ≥4 glasses daily) and how often they consumed candy (seldom/never, 1–3 times monthly, 1–3 times weekly, 4–6 times weekly, 1–2 times daily, ≥3 times daily).

### Statistical analyses

We calculated percentages, means and standard deviations for all included variables. The following background variables were dichotomized in the descriptive analysis (Table 1): Family economy into poor vs other, parental employment into full-time employment vs other, teeth brushing into ≤ once daily vs other, smoking habits into never vs other, spare-time physical activity ≥4 times per week vs other, daily screen time > 2 h daily vs other, educational plans > 12 years vs other, good grades into best or second-best grade in ≥1 of 4 subjects: Norwegian writing, mathematics, social science or English vs other, drinking sugar-containing soda ≥ daily vs other and consumption of candy ≥ daily vs other.

We calculated mean differences by using student’s t-tests, and risk differences by the cohort study command in STATA.

Data were analyzed using STATA 15.0 software (STATA, College Station, TX, United States: StataCorp, 2017). The 95% CI of the difference in various percentiles between the two time points was calculated using bootstrap resampling with 1000 replicates. The distributions were created with Epanechnikov kernel density plots in R Version 3.4.2. Vienna, Austria: R Foundation for Statistical Computing, 2017, www.R-project.org).

## Results

The mean age was 15.9 years (SD 0.3) in 2002 (*n* = 1675) and 15.8 years (SD 0.4) in 2017 (*n* = 1580). The proportions of boys were 50.9% in 2002 and 48.5% in 2017 (Table 1).

The 2017 cohort differed from the 2002 cohort in that more mothers worked full time, and that fewer parents smoked cigarettes. Further, fewer adolescents smoked and brushed their teeth ≤ once daily, but a larger portion sought help for mental health problems in 2017. More adolescents had screen time > 2 h daily, but more also participated in organized spare-time sports in 2017. There were more adolescents with a positive attitude towards higher education, and more adolescents achieved better grades and had plans for education beyond 12 years in 2017. Fewer adolescents consumed candy and sugar-containing soda daily in 2017 (Table 1).

The prevalence of OWOB increased by most background variables, including the sociodemographic variables, when comparing 2002 and 2017. (Table 1).

For girls, the mean BMI increased from 20.7 to 21.4 (mean difference 0.70, 95% CI: 0.40, 0.99, *p* < 0.001), while the mean BMI, at 21.5–21.4, was stable among boys (*p* = 0.80, Table 2).

**Table 2:** Mean anthropometric measurements of the participants, mean difference

	Girls		Mean difference, 95% CI	*p*-value	Boys		Mean difference, 95% CI	*p*-value
	2002	2017			2002	2017		
Height, cm	166.4	166.7			176.4	177.5		
Weight, kg	57.5	59.7			67.0	67.7		
BMI^a^	20.7	21.4	0.7 (0.37,0.95)	<0.001	21.5	21.4	−0.04 (− 0.35,0.27)	0.8
BMIz^b^	−0.07	0.22	0.29 (0.18,0.39)	<0.001	0.19	0.19	0.00(−0.10,0.10)	0.5

^a^Body Mass Index (BMI)

^b^BMI z- score (BMIz)

The prevalence of OWOB increased from 9 to 14% among girls (difference 5 percentage points%, 95 CI: 2, 8) and from 17 to 20% among boys (difference 3 percentage point, 95% CI: − 1, 6) (Table 3).

**Table 3:** Anthropometric measurements of the participants, risk difference

	Girls		Risk Difference, 95% CI	*p*-value	Boys		Risk difference, 95% CI	*p*-value
	2002	2017			2002	2017		
OWOB^a^ %	9	14	5 (2,8)	0.002	17	20	3 (−1,6)	0.18
OB^b^ %	1.8	2.5	0.7 (−0.7,2)	0.35	2.5	2.7	0.2 (−1,2)	0.73

^a^OWOB = overweight and obesity, age and sex-adjusted BMI > 25

^b^OB = obesity, age- and sex-adjusted BMI > 30

**Fig. 2:**
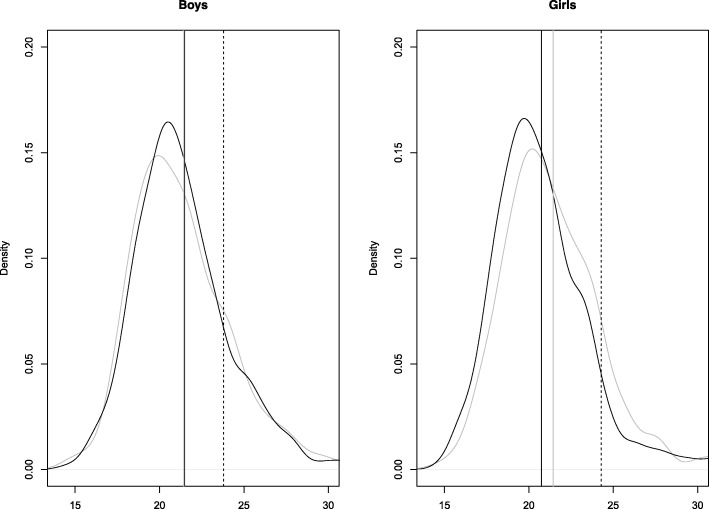
BMI distribution of boys and girls. BMI distribution of boys and girls.  (solid, vertikal black line) mean BMI (2002),  (solid, vertical grey line) mean BMI (2017)  (discontinuous, vertical line) OWOB (Overweight or obesity, age adjusted BMI > 25),  (solid black line in distribution) BMI (2002),  (solid, grey line in distribution) BMI (2017)

The shapes of the BMI density plots for both boys and girls were similar in 2002 and 2017 (Fig. 2). The mean BMIz increased significantly from − 0.07 to 0.22 (mean difference 0.29, 95% CI: 0.18, 0.39) among girls, while the numbers were stable at 0.19 (mean difference 0.00, 95% CI (− 0.10,0.10) among boys. For girls, a persistent mean difference in BMIz between 0.21 and 0.35 was found across all percentiles (5th -95th). For boys, mean differences per percentile ranged between − 0.06 and 0.09 (Table 4).

**Table 4:** Mean differences (change in BMIz from 2002 to 2017 by percentile)

	Difference	95% CI
Girls		
Percentile		
5	0.21	(0.02, 0.44)
10	0.31	(0.12, 0.51)
25	0.33	(0.19, 0.47)
50	0.29	(0.15, 0.43)
75	0.35	(0.19, 0.51)
90	0.30	(0.10, 0.51)
95	0.22	(0.15, 0.58)
Boys		
Percentile		
5	0.09	(−0.14,0.32)
10	−0.06	(−0.22, 0.11)
25	−0.05	(−0.20,0.09)
50	−0.03	(−0.14, 0.09)
75	0.06	(−0.08, 0.21)
90	0.00	(−0.18,0.17)
95	0.09	(−0.11,0.29)

## Discussion

The mean BMI and the prevalence of OWOB increased among Norwegian adolescent girls from 2002 to 2017. This change was due to an increase throughout the BMI distribution and is opposed to both our hypothesis and some previous findings [8, 9, 19]. No such change was seen for boys.

We found that the percentage of OWOB increased from 2002 to 2017 for almost all background variables, including the sociodemographic indicators. This finding is also supported by other studies [20] and it could be speculated that behavior has changed across socio-demographic levels towards a lifestyle favoring weight gain [21].

Public health promotion strategies and health-related habits are comparable between Norway and other European countries in many aspects. All children pay visits to the school nurse at 6, 8 and 13 years of age with additional visits for vaccines. The diet in Norway is generally varied [22] and adherence to nutritional guidelines among adolescents resemble that of other European countries [23]. Finally, the percentage of Norwegian adolescents meeting recommendations for daily physical activity corresponds to results from other European studies on adolescents [24] [25]. Still, the prevalence of OWOB is increasing among both Norwegian adolescents and adults [22], as in many other European countries [26].

Our finding of increased OWOB prevalence in girls is supported by a nationwide Norwegian report carried out during the same period [24]. There is a possibility that a sex-related increase in BMI appeared among boys before our study; mean weights for boys entering the military muster at age 17 increased between 1995 and 2008 and seemed to stabilize and decrease thereafter [27]. A regional study also revealed a higher BMI and an increasing prevalence of overweight and higher BMI values above the upper percentiles among adolescent Norwegian boys between 1966 and 1969 and 1995–1997 [28]. International, long-term studies of adolescents have shown mixed results; the mean BMI increased more among European girls than European boys between 1975 and 2016 [29], whereas global trends of OWOB between 1980 and 2013 displayed only small sex-related differences [30]. However, national and international trends in adolescent BMI and OWOB are difficult to compare due to low numbers of studies and differences in methodologies and results. This point is illustrated by the latter two studies where different growth-curves are used, resulting in different cut-points for overweight and obesity.

The average BMIs for girls in our study (20.7 and 21.4) are in the normal-weight range for both timepoints. Nevertheless, the increase in mean BMI is of clinical value, as changes in the mean value of a trait of a disease have established consequences for the frequency of illness [31]. Further, this will have consequences for the future prevalence of OWOB. Another important point is that the entire BMI-distribution for girls has shifted upwards on the BMI-scale from 2002 to 2017. This is underlined by the equal average increases in BMIz across percentiles for girls. This finding is concerning since girls, due to biological differences, gain increased fat mass compared to boys during adolescence [32].

For adolescent girls, our finding of an increased BMI throughout the total distribution may reveal a sex-specific obesogenic effect at the population level, and earlier studies have shown sex-related differences in weight gain due to both biological, behavioral and traumatic experiences [13, 33–35]. Due to the limitations of the cross-sectional design and the lack of other body measurements and biological tests, we were not able to explore changes in important risk factors that could explain the shift in OWOB and BMI scores.

Nevertheless, some perspectives regarding the increased BMI among girls seem relevant to consider. First, the adolescents in this study were exposed to the obesity epidemic both pre-, peri- and postnatally and were born prior to (1986) and at the height (2001) of a period of increasing birthweights in Norway [36]. A higher birthweight is correlated with an increased risk for later overweight [37, 38], although not with central adiposity or fat mass per se [39, 40]. The crossing of percentiles during the period from birth to adiposity rebound at 5–6 years of age has been seen as a critical period for later obesity, but might reflect increased growth in children that are already heavier instead [41]. Girls with higher BMI also tend to have earlier menarche, but the directionality of this relationship remains unclear [42]. In sum, children with high birthweight are vulnerable to subsequent higher BMI, but no clear pathway from high birthweight through adiposity rebound in pre-school age, early menarche and subsequent OWOB has been established.

Second, the obesity epidemic is a rather recent phenomenon that began 3 to 4 decades ago. Disentangling of the possible biological, societal, and environmental contributors to the etiology of obesity is ongoing. An example is the relatively newly gained knowledge of sex-specific increases in BMI and a higher risk of overweight in relation to dioxin exposure [43]. The main human sources of dioxins are foods, including meat, fatty fish, and dairy products, but dioxins are also concentrated in breast milk [44, 45]. We do not have a detailed record of food-intake, and therefore no measure of dioxin exposure in our study. Still, 90% of the adolescent cohort from 2017 had been breastfed, [46] and exclusive breastfeeding in Norway increased between 1998 and 2006 [47]. The possibility of breastfeeding as a mediator of adolescent OWOB contradicts the traditional view of breastfeeding as a protective factor from later overweight [48].

A strength of this study was that we explored the entire BMI distribution. This provides more extensive information than only BMI means or OWOB percentages. We used BMI, as this is currently the recommended screening test for obesity. We are not aware of any recent studies exploring secular change in BMI distributions in adolescents in other populations.

A notable weakness of our study was that height and weight were self-reported. We assume, however, that self-reporting may have reduced the number of refusals. A meta-analysis on self-reported BMI revealed an underestimation of the prevalence of overweight and obesity among girls and older children [49]. In addition, a Norwegian study found that adolescent girls significantly underestimated their BMI [50], yet with a high degree of agreement between self-reported and measured anthropometrics measured by intraclass correlation (intra-class coefficient for BMI was 0.87 in girls). On-line registration of self-reported height and weight has also been found to have high validity when compared to clinical examination [51]. This may imply a risk that our results underestimate the real BMI levels especially in girls, but likely so in both populations.

Another weakness is the lack of other metrics to explore overweight and obesity, i.e., waist circumference or percentage of body fat. BMI tends not to reflect percentage of body fat accurately [32], and especially among girls, an increase in waist circumference that is not explained by increase in BMI has been found [52].

Our study also lacks a measure of pubertal status. Females gain relatively more fat mass than boys during puberty and on average start puberty 2 years prior to boys. As the mean age of menarche in Norway has been stable at 13.2 years for the last 70 years [53] most girls in our study at both time points will have reached puberty. It is unlikely that puberty could explain the change in BMI for girls from 2002 compared to 2017.

The 2002 questionnaire lacked a date for when height and weight were measured, this may have led to modestly less precise calculations of BMIz. As both data-collections were conducted during the same months of the year, we again consider the datasets comparable.

A selection bias caused by a lower response rate among a larger group in the upper percentiles in 2017 cannot be completely ruled out. However, we have no specific indications of differences amongst the two groups of non-responders, and response rates of 80% (2002) and 70% (2017) are comparable to earlier observational studies on childhood OWOB [54].

We found sex-related trends in BMI and OWOB among Norwegian 15- to 16-year-olds. Girls had an increasing prevalence of OWOB and an increased mean BMI over the last 15 years represented by a uniform right shift in the entire BMI distribution. Thus, a shift of the entire BMI distribution in girls is explaining the increased prevalence of OWOB. Using OWOB to describe how a population is affected by an obesogenic environment accordingly has inherent limitations as the number of individuals above this cutoff vastly underestimates the number affected. Although the Norwegian rates of OWOB for children and adolescents are low compared to those in other European countries [26], we know that increasing BMI in late adolescence increases the risk of death from coronary heart disease in adulthood [1]. As cardiovascular disease is a common cause of death, especially in women, the impact of our observed trend on future health may be significant.

## Conclusion

We found that the increase in OWOB among 15–16-year old Norwegian girls presented a uniform shift in the entire BMI distribution, and was not due to a larger shift in a specific subpopulation in the upper percentiles. This finding may have significant implications on future health in Norwegian women.

## Data Availability

The part of the data collected in 2002 that support the findings of this study are available from the Norwegian Public Health Institute, but restrictions apply to availability of these data, which were used under the license for the current study, and so are not publicly available. Data collected in 2017 are however available from the authors upon reasonable request, and data collected in 2002 are available with permission of the Norwegian Public Health Institute.

## Abbreviations

BMI: Body Mass Index
BMIz: BMI z-score
OWOB: Overweight or obesity
